# Notes and News

**Published:** 1898-11-26

**Authors:** 


### Notes and News.

(Collected and Communicated.)

A French steamer brought a patient suffering from the plague to Delagoa Bay, but the Portuguese authorities refused to allow the ship to enter the bay. The same proceeding took place earlier at Mozambique. It is reported that the ship in question, the "Gironde," has sailed for Madagascar.

The trustees of the will of the late Mr. James Holden intend to devote £10,000 to the endowment of a wing at the Children's Hospital, Pendlebury; and the same amount is promised as an endowment to St. Mary's Hospital, Manchester. The Rochdale Infirmary has already received £18,000 from the trustees.

The India Office is inviting medical men to undertake temporary plague duty in Madras. Ten volunteers are asked for, and these will be required to start within two or three days' notice. Candidates must be under 40 years of age, and must forward a statement of their qualifications with their application to the India Office.

A Hospital for Women and Children has been opened at Jaffna, Ceylon. The funds were collected by the Misses Leitch, and the hospital handed over to the American Mission. At Inruville a hospital has also been opened under the American Mission. This latter institution has been placed in charge of Doctor Isabella Curr. At Galle it is decided to station a hospital ship for the treatment of plague cases.

We learn that, following the appeal made by Mr. F. A. Bevan on the 15th inst. for £12,000 to complete the British Home for Incurables, Streatham, by the addition of an entertainment hall and further accommodation for patients and staff, the Home being now full, a lady, who wishes to be anonymous, has handed to the Secretary of the Institution, at 72, Cheapside, the sum of £1,000, for the purpose of endowing a bed in the new wing in memory of her mother, and to be known by the initials "A. M. R." It is earnestly hoped that this example may be followed by the endowment of the five other beds which are to be added to the seventy now occupied at the Home.

The Charing Cross Hospital has received £41,000 towards the £100,000 for which it has appealed to effect necessary improvements and additions. The latest contributions included one of £1,000 from "H.," and one of £500 from "G."

The meetings of Irish guardians are usually marked by some amusing incidents. It would be more amusing if the subjects concerned were not of a serious nature. For instance, at Ballymena the matron complains of the diet ordered for typhoid patients in the Fever Hospital. Confronted with this complaint the Board of Guardians remember the Local Government Board, and solve their difficulties by asking the Local Government Board to hold a sworn inquiry. The master's difficulties in removing fever patients is also to be carried to Olympia, only one guardian protesting, who said he thought too much was being made of the fever.

The importance of furthering the study of tropical diseases amongst English medical men is being undoubtedly recognised. A few years hence and English people will be surprised that the necessity was so long unrecognised by a nation who sends so many of its population to unhealthy tropical regions. At the annual dinner of the Royal Southern Hospital, Liverpool, Professor Paterson proposed that a scheme such as is proposed at the Seamen's Hospital, Greenwich, should be adopted at Liverpool, and it was announced that money had already been contributed for the purpose. Is Liverpool going to occupy the van in this progressive movement?

Last year the opponents of the Hospital Sunday Fund, and there are unfortunately several, maintained that it was a dying organisation, because in certain places the amount received that year on Hospital Sunday showed no increase over or was less than last year. It is interesting, therefore, to notice that in Birmingham on Hospital Sunday this year 50 congregations collected £2,869 for the General Hospital, whereas 55 places of worship in 1895 contributed only £2,342, thus showing an increase in the present year over 1895 of £527 from five fewer congregations. At Newcastle, too, it is pleasant to notice that the second list of the Sunday collections there shows an increase of £210 9s. 8d. as compared with the corresponding list of last year.

The reports from Central Asia show that the outbreak of plague which was notified some time ago has been of a very serious character. Down to November 4th, out of 357 inhabitants of the village of Anzob 233 had died, and 14 remained on the sick list. Very elaborate precautions have been taken to prevent the contagion spreading beyond this village, to which it so far seems to have been restricted. A supply of serum has been obtained, and preparations have been made for inoculating those likely to be exposed to infection. In the principal Russian towns 100 doctors and 80 assistants are already registered and ready to start for their appointed places at a moment's notice. Posts of medical observation have been established in three lines of towns and villages surrounding the seat of the outbreak, and others on the various lines of communication by river and by railway, on the Caspian and in the mountain passes. Troops, as well as the inhabitants, are being employed in forming cordons of isolation.

